# Transforming the nursing curriculum through required interprofessional education: key issues for consideration

**DOI:** 10.4069/whn.2026.06.13

**Published:** 2026-06-30

**Authors:** Gayle M. Timmerman, Veronica Young

**Affiliations:** 1 School of Nursing, The University of Texas at Austin, Austin, TX, USA; 2 Center for Health Interprofessional Practice and Education, The University of Texas at Austin, Austin, TX, USA

## Introduction

In earlier periods, when health care knowledge was relatively limited, individual health care providers could often provide adequate patient care on the basis of their own knowledge and experience, with little need for collaboration. However, the rapid expansion of scientific knowledge has led to substantial advances in health care, including evidence-based treatments, diagnostic procedures, and medications. As knowledge has expanded and care has become more complex, individual health care providers from a single discipline can no longer provide high-quality, comprehensive care alone. These changes have moved health care toward interprofessional, team-based collaboration [1-3].

Within this context, improving the quality of care has become a central priority. In 1999, for example, the Institute of Medicine (IOM) issued a call to action to improve health care safety by reducing medical errors [4]. Deaths attributable to errors have been estimated to reach as many as 250,000 annually, and most serious health care errors have been linked to failures in team communication [5,6]. Improving safety requires moving beyond a focus on individual mistakes and examining the human and system factors that contribute to errors, including breakdowns in processes and communication [4]. Thus, how care is delivered, including how teams communicate, is as critical as the care itself [7].

More broadly, the IOM proposed six domains for improving care quality across the continuum from inpatient care to community health care: care must be safe, effective, patient-centered, timely, efficient, and equitable [8]. Redesigning health professions education is central to creating a safer health care system, particularly by preparing learners for interprofessional, team-based care that is evidence-based and focused on quality improvement [3]. In 2011, the Interprofessional Education Collaborative (IPEC) provided the first roadmap for defining the competencies essential to collaborative practice and team-based care, setting health professions on a path toward integrating interprofessional education (IPE) into core curricula [7].

The nursing profession represents the largest segment of the health care workforce, placing nurses in a key position to influence care quality and patient outcomes [9]. Because nurses spend more time at the bedside and in direct contact with patients than many other health professionals, they are essential members of health care teams and help ensure safe, high-quality, person-centered care [10]. Accordingly, advancing professional nursing education requires IPE that prepares nurses to coordinate care effectively, engage with providers from other health professions, enhance team functioning and patient safety, and assume leadership roles when needed [9-11].

Nevertheless, despite evidence that effective collaboration among health care teams improves health outcomes [2], interprofessional collaboration and team-based care have not been consistently realized across health care settings [12]. To address this gap, IPE has been promoted as an alternative to isolated, siloed education within individual health professions. Its purpose is to develop the competencies needed for interprofessional collaboration and to prepare graduates for collaborative practice as they enter the workforce [6,13]. In a recent scoping review, Cadet et al. [2] found strong support for a positive association between IPE and clinical health outcomes, including length of stay, medical errors, and patient satisfaction.

At a global level, approximately half of academic institutions report having established IPE programs, and one-third require IPE for all students; nursing is the most frequently represented profession [14]. Given the continuing need for international growth in IPE, a clearer understanding is needed of the factors that facilitate IPE programs, especially among institutions beginning their IPE development. In this article, we examine key issues for programs planning to transition to required IPE, along with useful resources and examples from our experience at The University of Texas at Austin.

IPE “occurs when students from two or more professions learn about, from, and with each other to enable effective collaboration and improve health outcomes” [15]. This definition is widely accepted and allows flexibility in the professions that learn together and the number of professions that must interact. In universities, this flexibility is logistically useful because the health professions programs available for IPE vary across institutions. For example, in our Center for Health Interprofessional Practice and Education (HIPE Center) at The University of Texas at Austin, we include all of our health professions programs—not only nursing, pharmacy, social work, and medicine, but also clinical and counseling psychology, audiology, speech-language pathology, and dietetics. Each university has its own configuration of health professions programs, and this configuration shapes the design of its IPE experiences and programs.

The preceding definition also clarifies what does not meet the criteria for IPE. IPE requires interaction among students from different professions that supports collaboration through shared team goals, accountability, and decision-making [13]. For example, if nursing and medical students take an ethics course without planned interaction or opportunities to learn from one another, the course does not constitute IPE. Although clinical training in hospitals is often described as IPE, these experiences also fail to meet IPE criteria when students from different health professions do not actually collaborate, which does not consistently occur in clinical settings. In hospitals, for example, nursing students may observe physicians’ interactions with patients without participating in those encounters. Because the nursing student and physician in this example do not interact directly, the situation does not represent IPE; the participants cannot learn about, from, and with one another.

## IPEC Core Competencies

The development of IPE was closely tied to the formation of IPEC, which currently includes 22 educational associations representing different health professions, including nursing, medicine, pharmacy, dentistry, public health, social work, and dietetics [13]. In 2011, IPEC released the first Core Competencies for Interprofessional Collaborative Practice to guide IPE curricular development. After a rigorous revision process that incorporated input from key stakeholders and drew on more than 10 years of experience implementing the original IPEC Core Competencies in education and practice, IPEC released Core Competencies for Interprofessional Collaborative Practice: version 3, which is available online at no cost [13]. In this refined version, four main competency categories remain: (1) values and ethics: “work with team members to maintain a climate of shared values, ethical conduct, and mutual respect”; (2) roles and responsibilities: “use the knowledge of one’s own role and team members’ expertise to address individual and population health outcomes”; (3) communication: “communicate in a responsive, responsible, respectful, and compassionate manner with team members”; and (4) teams and teamwork: “apply values and principles of the science of teamwork to adapt one’s own role in a variety of team settings” (p. 16–19). Each competency is accompanied by 5 to 11 subcompetencies that further clarify expectations. IPEC also clarifies that patients, their families, and/or the community are team members [7].

The use of these competencies provides a common language and clear expectations for interprofessional teamwork across professions, thereby offering a framework for improving health care delivery and health outcomes [13]. The IPEC Core Competencies have been endorsed by multiple health professions accrediting agencies, which has contributed to their widespread adoption by diverse health professions and educational institutions across the United States [13]. The IPEC Core Competencies are central to the design of both IPE programs and individual IPE learning activities.

## Transition to the required interprofessional education

Because interprofessional practice and education are considered critical to patient safety in health care systems [4,16], most health care professions require the development of interprofessional competencies to meet accreditation standards [17]. The addition of IPE to accreditation standards has provided an impetus for programs to identify innovative ways to make IPE a required component of their curricula. In a 2019 survey from the American Interprofessional Health Collaborative [13], 92% of IPE leaders across the United States reported that some type of IPE was required for all or some students. This finding demonstrates the growth of IPE since the release of the original IPEC Core Competencies, with IPE now included as a mandatory accreditation standard in the United States for nursing, medicine, and pharmacy.

The American Association of Colleges of Nursing (AACN)’s Essentials: Core Competencies for Professional Nursing Education drives curricular requirements for nursing accreditation in the United States [11]. This document presents 10 domains, or areas of competence, with corresponding competencies and subcompetencies that represent the core of nursing practice. Although the domains and competencies are the same across prelicensure, entry-level, and advanced-level graduate nursing education, the subcompetencies reflect different expectations for each learner level.

To advance IPE by improving person-centered care and population health outcomes, Domain 6 of the AACN Essentials focuses on “Interprofessional Partnerships: intentional collaboration across professions and with care team members, patients, families, communities, and other stakeholders to optimize care, enhance the health care experience, and strengthen outcomes” [11]. The four competencies in this domain are consistent with the IPEC Core Competencies, to which the AACN contributed, and reflect nursing’s distinct contribution to the health care team, as well as nurses’ knowledge of other professions’ roles and responsibilities. These competencies address interprofessional collaboration, communication, leadership, and team dynamics [11].

In addition, the nursing competency “Provide Care Coordination,” found in Domain 2, Person-Centered Care, highlights the nurse’s role in facilitating continuity of care, coordinating care in collaboration with the health care team, and promoting collaboration by clarifying responsibilities among team members [11]. Effective care coordination occurs when each profession understands and uses the expertise of other professions to support person-centered care [7]. This curricular requirement substantiates the key role of nurses on interprofessional health care teams. Accreditation requirements within and across professions therefore play an important role in promoting high-quality IPE [17].

Before the release of the IPEC Core Competencies and the AACN accreditation mandate, IPE in nursing programs often consisted of optional elective courses, isolated events, or incidental encounters. Organized early IPE initiatives, such as elective courses, had the advantage of attracting faculty and students who were self-selected, motivated, and interested in developing collaborative relationships with other professions [18]. For example, one of our early IPE efforts at The University of Texas at Austin was an elective course that gave graduate students in nursing, medicine, pharmacy, and social work the opportunity to work collaboratively on interprofessional teams. These teams developed proposals for learning activities designed to foster IPE competencies, such as medication reconciliation and team error disclosure. Student feedback was very positive, with students commenting on the value of working as a team and developing a greater appreciation for members of other professions.

Offering an elective worked well for the limited number of students who made IPE a priority in their schedules. However, given the increased emphasis on IPE in accreditation requirements and its importance for safe, high-quality health care, IPE should be required for all health professions students, not only those already committed to interprofessional collaboration. Students least interested in IPE may be the ones who need it most. Integrating IPE experiences into the curriculum creates challenges related to scalability, logistics, resources, and student and faculty motivation. At the same time, it creates opportunities to develop leaders who can transform health care and improve patient safety and care quality.

## Institutional support for quality IPE

Faculty need time to develop IPE across their respective professions and to make curricular changes, and this work requires institutional support. To overcome obstacles to IPE, institutions should prioritize IPE; establish a centralized, institution-wide structure for organizing IPE across professions; and fund staff positions and faculty release time to coordinate and facilitate IPE [17]. Globally, approximately two-thirds of academic institutions report having centralized IPE, although substantial regional gaps remain [14].

In the United States, centralized IPE has often been implemented through IPE centers, institutes, or offices. These entities vary widely in organizational structure, location, funding mechanisms, budget, leadership structure, and dedicated staff and faculty time. IPE centers, institutes, and offices are typically accountable to all partnering health professions on campus and to the central administration, and they provide leadership, coordination, and staff support for IPE [19].

The addition of IPE to accreditation standards provides justification for the institutional resources needed for IPE development, implementation, and sustainability. Because institutional support is essential, Zorek et al. [20] developed the IPEC Institutional Assessment Instrument to assess institutional characteristics that may serve as barriers to, or facilitators of, successful implementation of high-quality IPE programs. The instrument identifies multiple institutional characteristics clustered around three factors: (1) institution-wide infrastructure supporting IPE, such as IPE centers, institutes, or offices; (2) institutional commitment to IPE, such as long-term commitment from leaders and faculty across professions who value IPE; and (3) use of the IPEC Core Competencies as a framework, such as promoting IPEC competencies to guide activities of increasing complexity across the curriculum and to meet the needs of students from multiple health professions. Zorek et al. [20] noted the importance of having a designated leader with budgetary oversight to sustain long-term institutional commitment to high-quality IPE.

Although the IPEC Institutional Assessment Instrument is useful for internal institutional planning and quality improvement [20], its global use has been limited, and adaptation may be needed for use in different countries. Regardless, the instrument’s items and expert consensus statements address institutional issues that should be considered when launching or improving IPE programs. The IPEC Institutional Assessment Instrument can be downloaded at no cost from https://www.ipecollaborative.org/ipec-institutional-assessment-instrument.

A lack of strong institutional support does not preclude the development and implementation of IPE. Many IPE programs operate despite limited resources. To compensate for minimal institutional support, some health professions programs share the costs of IPE. Enthusiastic and committed faculty champions can also drive IPE programs, even in the absence of formalized support. Educational and training resources for IPE can be obtained from international and national organizations (Table 1). However, minimal institutional support reduces the resources available and limits the coordinated leverage across programs that can be used to overcome barriers to sustainable IPE [10]. Administrative support is needed to prevent IPE from overburdening IPE champions who already have full-time responsibilities.

## Key issues to consider in designing the required IPE

When designing required IPE, the goal should be to develop longitudinal plans that build competency expertise across the curriculum while progressively increasing complexity [13]. High-quality IPE should be intentional and should consist of a series of IPE experiences spanning the curriculum that move students toward mastery of the IPEC Core Competencies [17]. By deliberately working with students from other health professions toward the shared goal of improving person-centered and population-oriented care, students learn to maintain a climate of mutual respect based on trust, honesty, and integrity; understand each health profession’s role in delivering person-centered care; communicate effectively with patients and other health professionals; and function as effective team members by applying principles of team dynamics [13].

Planning typically begins with faculty committees or task forces representing the professions that will participate in IPE. These professions may have different processes for curriculum change, which can affect implementation timelines. Sustainable IPE programs require each health profession to integrate IPE competencies into its respective curriculum. Planning must also include frequent assessment of learners’ competencies, continuous quality improvement, and program evaluation.

Consistent with best practices for managing systems change, theories of change should guide this process. Change requires buy-in from leaders and key stakeholders to minimize resistance [6]. A clear sense of urgency and a well-articulated rationale for shifting from traditional education to IPE are needed to offset the effort required for change [6]. This process can be especially complicated when multiple health professions programs must change simultaneously.

Because IPE requires active participation from students across professions, common curricular threads among programs, such as communication skills and ethics, may provide efficient opportunities for faculty to plan joint learning activities. For example, the HIPE Center conducted a needs assessment in its first year to determine how best to organize IPE across professions and prioritize the most relevant topics. The joint topics selected for IPE included team error disclosure, working with patients with substance use issues, discharge planning, and disaster preparedness.

It may be easier to implement a longitudinal plan in phases rather than introduce multiple curriculum-wide changes at once. For programs beginning IPE for the first time, starting with a single IPE activity is recommended [10]. Lessons learned from piloting that activity, along with the interprofessional faculty relationships developed during the process, can inform plans for standardizing IPE in the curriculum. Several IPE toolkits provide practical guidance for IPE program design, implementation, and activities [6,10].

During early planning, it is also necessary to identify the learner levels in each profession and develop a longitudinal plan for building competencies as students progress through their programs. We began by planning a required Foundations of Interprofessional Collaborative Practice (FICP) course designed to introduce IPE to learners early in their curricula; we then added IPE experiences in which students could demonstrate application and competence. Our FICP course required 2 years of intensive effort, from planning through implementation.

## Tailoring IPE to the health professions involved

Each profession engaged in IPE must have a role in designing and implementing interprofessional team activities, such as case studies and simulations [17]. For example, when we expanded our IPE program to include health professions that had not previously participated in IPE, we chose content on team communication in health care settings that was relevant to all participating professions. We used communication strategies from TeamSTEPPS (Team Strategies & Tools to Enhance Performance and Patient Safety) in small-group team activities. TeamSTEPPS is an evidence-based teamwork system designed to improve patient safety by strengthening interprofessional communication and teamwork [21].

IPE planning should ensure that faculty from the participating professions provide input so that each profession’s perspective and contribution to the team are represented. When a faculty member from one profession adds another profession’s role to a case or simulation, the interprofessional activity may remain effectively uniprofessional if there is no shared understanding of expected team performance; this can risk perpetuating professional biases and stereotypes [6]. For example, when we developed videos to illustrate the application of the IPEC Core Competencies with nursing, medicine, pharmacy, and social work represented on the team, we quickly found that faculty from each profession needed to develop the content for their respective roles to maintain authenticity and accuracy.

In planning IPE activities, we learned early that failure to clearly communicate the value of each health profession’s contribution to each activity led to student dissatisfaction with the IPE experience. For example, students emphasized the importance of seeing their own profession represented on interprofessional practitioner panels.

## Tailoring IPE to learners’ levels

IPE should also be tailored to learners’ levels [17]. Learners should be at similar levels, or more advanced learners should be given additional challenges. For example, our 3-hour virtual Interprofessional Community Health Intervention Planning simulation, in which an evidence-based improvement process guides a community health intervention addressing adolescent vaping, includes two levels of interprofessional experience: (1) students who work in interprofessional teams and (2) graduate students with greater health care experience who receive additional training to serve as team facilitators. This structure gives more advanced students interprofessional experience in leadership and facilitation while providing the program with additional facilitator resources to run the simulation for less experienced learners [22].

### Dual-identity development beginning with early learners

Preparing learners for interprofessional collaborative practice requires the concurrent development of professional and interprofessional identities, a process referred to as dual-identity development [17,23]. Introducing IPE early in the curriculum supports this developmental process. However, designing learning experiences for students who have not yet developed a clear understanding of their own professional role presents a challenge. The second IPEC core competency, roles and responsibilities [13], emphasizes that students must use knowledge of their own roles and those of other team members to improve health outcomes. Addressing this competency before students have formed a professional identity requires careful, intentional curricular design.

During initial implementation of the FICP course, early learning activities emphasized team-building and communication while de-emphasizing clinical knowledge and understanding of professional roles and responsibilities. This approach reflected concern that most students were still in the early stages of professional identity formation. Our learners included first-year pharmacy and medical students, undergraduate nursing students, and undergraduate and graduate social work students. End-of-semester student feedback, however, indicated the need for a more explicit connection between team-building activities and clinical scenarios to increase relevance to future practice. Students expressed a strong preference for teamwork applied to clinical scenarios. Additional feedback highlighted the value of role-play and simulations, small-group discussions, and real-world experiences shared by faculty facilitators.

To increase the clinical application of interprofessional competencies for students without established professional identities, we added several teaching strategies. Interprofessional practitioner panels provided relevant clinical examples and illustrated interprofessional teams in practice, helping bridge the gap between knowledge and practice for students early in their programs. Faculty facilitators also shared practice experiences during team time, which further strengthened the focus on clinical application.

Role-play and simulation were the learning strategies that students found most useful. Students recommended that these strategies be introduced early in the course and used as frequently as possible. For example, students in the FICP course participate in a team discharge planning meeting using a case study in which they play their respective professional roles. Because students are early in their curricula, we provide information about the discharge planning tasks to be completed, and each profession receives a separate handout outlining its role in that process. Students use the TeamSTEPPS Brief Checklist tool to role-play the discharge planning meeting, with instructions to develop a discharge plan and negotiate its implementation, including who will do what [21]. Evaluation data from the session indicated that students agreed or strongly agreed that the session provided opportunities to learn about one another’s professions (89%) and promoted collaboration with other professions (93%). One first-year student who participated in the activity commented that the session “helped me understand my role better and gave insight into how to work among other health care providers.”

Lessons learned included that early learners value interactive, clinically relevant, role-specific learning activities that improve their understanding of their own roles and responsibilities, as well as those of other health care team members. Even when students have not yet developed professional role identities and have limited health care knowledge, they can successfully practice interprofessional communication and teamwork if they are given information about their profession’s expected role in the scenario and relevant health care information is presented without medical jargon.

### Challenges with perceived value and the applicability of IPE

Evidence from systematic reviews indicates that IPE generally supports the development of learners’ knowledge, skills, and attitudes [24]. For IPE to be prioritized, it must effectively develop the competencies required of students. In addition to the issues discussed above, effective IPE is influenced by (1) readiness for interprofessional learning, (2) skilled facilitators who provide guidance and support reflection, and (3) real-world clinical settings or cases that facilitate team-based experiential learning [25].

Students have demanding, rigorous curricula that require substantial time and commitment. Without an explicit explanation of the purpose, value, and relevance of IPE to clinical practice, some students have reported viewing IPE as unnecessary or of limited value [25]. In addition, attitudes among students and teams regarding the value of IPE and teamwork are intermediary factors in IPE effectiveness; those who perceive IPE as beneficial are more likely to achieve IPE competencies [25].

In a review of the literature, Bogossian et al. [18] identified several factors that can affect students’ development of competence in interprofessional collaboration: perceptions of unequal role status, hierarchical barriers, negative stereotypes, preconceived biases, learner resistance, lack of buy-in to IPE, and skepticism. Noureddine et al. [6] found that after a culminating IPE event, students reported a high level of interprofessional prejudice; more than 30% were biased against other professions, and more than 30% perceived that other professions were biased against them.

These issues should be acknowledged thoughtfully and addressed in IPE planning. Noureddine et al. [6] recommend that all IPE activities include discussion and learning that promote awareness of implicit biases against other professions, because such awareness is a first step toward correcting misconceptions. IPE activities that provide opportunities for students to interact with peers from other professions can help reduce preconceived biases [6]. Faculty facilitators can also play a critical role in addressing bias by exploring biased comments nonjudgmentally to better understand and correct misperceptions.

Not all attempts to address stereotypes and bias will work. For example, one of our IPE activities for the roles and responsibilities competency used drawing to explore stereotypes by profession. Some students felt that addressing stereotypes in this way perpetuated, rather than reduced, professional stereotypes.

On the basis of our experience, even when most IPE learners are motivated and accept the value of IPE, faculty and/or facilitators need to do the following for each IPE activity: (1) clearly articulate the value of team-based practice, (2) clarify the role of the specific IPE activity in developing IPE competencies, and (3) explain how the IPE activity applies to practice. IPE and clinical topics should be presented as equally important [18]. Faculty should be transparent about why learners need to engage in IPE and develop IPE competencies, and they should explain how this content is relevant to clinical practice to strengthen individual learner motivation. Guided debriefings after IPE activities may also help students connect the learning experience to practice and better understand its applicability.

Lack of engagement during IPE activities may reflect a student’s low perceived value of IPE, or it may simply reflect personal distractions. To address inattention, such as the use of electronics unrelated to class, individual teams can develop their own ground rules for group behavior and hold one another accountable [26]. Faculty facilitators can help keep students focused and emphasize the importance of the activities. Given the effort required to coordinate IPE, each intentional encounter with other professions should be treated by students as a valuable learning experience to be fully used.

### Solving logistical issues

Some of the most challenging issues in IPE are logistical. A solid IPE plan should be in place before implementation. Addressing logistics requires knowing which professions are involved, the learner levels in each profession, the number of students from each profession, students’ schedules, the number of faculty members and facilitators needed, the setting for IPE (e.g., online or in-person classrooms, clinical settings, or community settings), and any additional resources required, such as classroom supplies or standardized patients. Logistical challenges are significant threats to IPE implementation and must be addressed well in advance through regular coordination among administrators, faculty, and support staff. These meetings should ensure that participants from different programs, including those who can facilitate scheduling and adjust curricula, are engaged and have reached consensus [18].

Scheduling IPE experiences at times that accommodate multiple professions’ schedules, finding sufficient space for large numbers of students from multiple programs, and providing and communicating consistent expectations and incentives for diverse learners are all logistical challenges. For our FICP course, the only mutually available time that we could negotiate among the nursing, medicine, pharmacy, and social work programs, while avoiding weekends and evenings, was Fridays from 2 to 5 p.m. Some virtual interprofessional simulations in our microcredential program are scheduled on Saturday mornings.

For IPE, in-person classroom and online settings can provide useful learning environments for practicing teamwork, interprofessional communication, and problem-solving [18]. Delivering these experiences to large numbers of students, however, requires additional planning. To accommodate large numbers of students in our FICP course, ranging from 296 to 375 students, we divided students into three cohorts, each consisting of 12 interprofessional teams of seven to nine students and one to two faculty facilitators. Each team attends six 3-hour sessions during the semester. All 36 teams attend the opening session concurrently; thereafter, one cohort meets each week for the same content module before moving to the next module, rotating through each class module during the semester. For example, the Team Addiction Care module is taught for 3 consecutive weeks, with a different cohort attending each week.

Dividing student teams into three cohorts creates a more manageable learning environment, with less classroom noise and greater personalization, and maximizes the use of resources. It is easier to recruit interprofessional faculty facilitators when they are assigned to the same team and required to facilitate six sessions per semester rather than weekly sessions; this structure better accommodates busy practice schedules and encourages involvement. We have been able to maintain a consistent core of dedicated interprofessional faculty facilitators, who serve as role models. The cohort model with small groups also worked well when the course moved online during the pandemic, with breakout rooms used for small-group work. However, online platforms may limit the number of students who can participate, and these limits must be considered in planning. In student feedback for FICP, learners valued team interactions and preferred that some class time be spent with their teams in small-group rooms rather than in the larger classroom. Individual small-group rooms allow for better team interaction, greater privacy, and more in-depth discussion away from the noise and movement of the large group.

Once each semester, the FICP course includes an event during class time that expands beyond the enrolled students’ fields to include additional campus health professions, such as dietetics, audiology, and speech-language pathology. Students from these additional health professions are not enrolled in the course; instead, they receive microcredentials, or digital competency-based educational certifications, for their participation and preparatory work. To accommodate the additional students, the small-group teams are reconstituted and expanded from 36 to 47 teams, with approximately 400 learners. Campus space that can accommodate this number of in-person learners seated at tables in groups is very limited; large lecture halls with fixed stadium seating are not suitable. We ultimately rented a ballroom in one of the student activity centers to accommodate this activity. This required additional resources beyond those typically needed for other courses.

The logistical challenges differ for each IPE program and activity. To address these challenges, planning must begin early. Because each profession has unique issues that affect logistics, the professions involved in the planned IPE activity must work together to find solutions that meet everyone’s needs. Compromise is often essential for solving IPE-related logistical challenges. Institutional support may also provide resources that help address logistical barriers.

## Structuring IPE experiences

IPE can be delivered through stand-alone courses and/or embedded within existing courses, which may be didactic or clinical. The defining feature of IPE experiences is that learners develop IPEC Core Competencies “when students from two or more professions learn about, from, and with each other to enable effective collaboration and improve health outcomes” [15]. Consistent with competency-based education, IPE programs should intentionally move learners from introduction through practice toward mastery. This progression requires frequent assessment and self-assessment, feedback, and opportunities for practice [11].

Didactic IPE works well for introducing the IPEC Core Competencies to interprofessional learners. Typically, interprofessional faculty deliver theory bursts, defined as brief segments of lecture content, followed by application activities completed in small interprofessional teams [6]. These application activities must include enough time for students to learn about, with, and from one another [15]. Interprofessional faculty facilitators on these teams are responsible for (1) guiding interprofessional communication and teamwork, (2) clarifying expectations, roles, and responsibilities across professions and their application to clinical practice, and (3) facilitating reflection [26].

Clinical IPE experiences include high-fidelity simulations, community-based clinical experiences such as vaccine clinics and health fairs, and interprofessional clinical practice activities such as team meetings and clinics [6]. Simulations and clinical practice activities may be embedded in students’ clinical courses. For example, we include a hospital simulation day in our simulation center as part of undergraduate nursing students’ adult health clinical course. These nursing students are assigned hospitalized patients, with another student playing the role of the patient, to practice nursing care as the case unfolds. The simulation becomes interprofessional when social work students collaborate with nursing students to address patients’ needs from a social work perspective or when pharmacy students take calls during the simulation to consult with nursing students about medication-related questions and problems.

IPE programs across academic institutions vary in structure, delivery mode (in-person, online, or hybrid), type of IPE experience (e.g., case studies, simulations, role-play, games, projects, clinical practice, or community service), scenario or setting (e.g., community-based integrated care or acute care), and use of incentives (e.g., grades or microcredentials). The effectiveness of IPE is strengthened by (1) problem-based learning that provides authentic learning activities, such as real-life scenarios, and promotes collaboration; (2) experiential learning that uses hands-on active strategies with reflection, such as role-play and simulations; (3) community-based learning that engages students in authentic societal issues within the community, such as student-run clinics and home visits; and (4) technology-based learning that incorporates technology into team-based learning, such as virtual escape rooms and interactive computer simulations [25]. Team-based learning is more effective than large-group discussion and more closely meets the definition of IPE [18].

The mode of delivery—in-person, online, or hybrid/blended—may vary depending on needs and circumstances. Some IPE activities, such as collaborative practice activities and simulations involving hands-on skills, must be delivered in person. Online IPE can avoid many logistical barriers associated with in-person IPE, including geographic barriers, the need to collaborate with programs that are not located on the same campus, scalability constraints, limited physical space, and cost [6]. Physical space may be especially challenging when available rooms must be suitable for teamwork, conveniently located for different professions, and large enough to accommodate the required number of teams. Scenarios designed to train interprofessional student teams for telehealth may be particularly useful as online IPE experiences, because telehealth is now used in more than 60% of US health care institutions [6].

Effective online learning can deliver foundational knowledge about teamwork, communication, and different professional roles and responsibilities, leading to positive attitudes and new knowledge [6]. In comparisons of online and in-person IPE delivery, students have reported similar benefits for both formats [27]. However, qualitative data indicated that many students preferred in-person delivery, which they viewed as more valuable, more engaging, and more conducive to learning from team members [27].

The type of health care setting chosen for IPE varies according to the professions involved and setting availability; available settings may differ by country, health care funding, and geography. Regardless of setting, key curricular components include meeting the IPE definition [15] and building IPEC Core Competencies [13]. For example, an IPE activity could be designed around the health professions that collaborate in community-based integrated care settings, such as social workers, public health professionals, care managers, nurses, physical and occupational therapists, mental health specialists, and community welfare personnel. In this setting, students could learn about the roles and responsibilities of these health care professions while learning about, from, and with one another. At least two professions, such as nursing and physical therapy, would need to interact. The IPE activity could occur in a clinical setting, such as during home visits, or the setting could be used as the basis for a case study or simulation.

When structuring IPE experiences, it is useful to consider incentives for team members from different professions. Examples include grades, microcredentials, credit for clinical or laboratory hours, volunteer hours, and/or personal development. Ideally, incentives should be uniform across all students on the team. However, this is not always possible when the same IPE activity is embedded in different courses and incentives are controlled by the faculty who teach those courses. In most cases, this is not a major issue. However, conflict can arise in a group project if students from one profession receive a grade while students from another profession receive only participation credit. Students who are not graded may be less motivated to contribute because of competing priorities, which can leave graded students with additional work. In such a scenario, faculty could change the project to participation grades for all team members, have ungraded students serve as consultants with redefined team roles, or ask the team to discuss the incentive disparities transparently and negotiate accountability for each member’s project deliverables.

### Microcredential/badging structures for IPE

One challenge in designing an IPE program is determining how to structure educational offerings to meet evolving learner needs, including the distinct requirements of different health professions programs and the development of interprofessional competencies. Microcredentials have emerged as a flexible approach to learning that is intended to respond to changing workplace skill needs. Unlike traditional degree programs, microcredentials, sometimes referred to as badges, are designed to be modular, accessible, rigorous, and skills-based. This modular design supports personalized learning pathways aligned with individual career development goals. Such a framework aligns closely with competency-based education, in which learners demonstrate skill development. Its use is increasingly common in higher education institutions, including those in the health sciences [28]. Examples of institutions offering IPE microcredentials include the University of Wisconsin–Madison, Texas Tech University Health Sciences Center, Quinnipiac University, and the University at Buffalo.

At our institution, we adopted a microcredential framework to design learning pathways that help learners in the health professions develop the skills and IPEC competencies needed for effective interprofessional collaborative practice. Because IPE requirements are embedded across several program-specific courses, the microcredential pathways provide an intentional structure that enables learners to recognize and track the development of their interprofessional competencies and other collaborative skills. Our microcredential program is led and coordinated by the HIPE Center, with input from the Executive Steering Committee and the IPE Curriculum Committee, which includes faculty representation from all health professions programs. The program is supported by university-provided staff and software and aligns with the university-wide digital badging initiative. All IPE microcredential proposals undergo a rigorous quality review process and must be approved by the IPE Curriculum Committee before being submitted to the university for final approval.

Our microcredential program consists of three levels of experience: foundation, application, and demonstration. Foundation-level IPE experiences, intended for early learners, are integrated into required courses across most health professions programs and emphasize early dual-identity formation, team communication and teamwork strategies in health care practice, and an interprofessional approach to increasing access to care. The application level, intended for intermediate and advanced learners, comprises a curated and expanding menu of approved interprofessional experiences designed to give learners flexibility in selecting activities aligned with their interests and program-specific requirements. The demonstration level serves as a capstone experience and consists of a simulation in which advanced learners from each program apply profession-specific knowledge while demonstrating proficiency in the IPEC Core Competencies. Currently, most programs require students to complete the foundation and demonstration levels while encouraging engagement in application-level experiences offered at the course and/or program level. This structure, within the microcredential framework, encourages faculty with shared expertise across professions to co-develop high-quality IPE experiences for learners.

## Faculty development and recruitment

As part of IPE planning and implementation, faculty development plans are needed to build skills in facilitating teams and teamwork, as well as skills in helping students understand and meet the IPEC Core Competencies. For some faculty, working across disciplines may be a new experience, and they may also need to strengthen their own interprofessional collaborative skills. Because IPE activities require faculty from multiple professions with varying levels of IPE experience, adequate faculty training should be provided for each IPE activity to ensure high-quality learning experiences [18]. IPE experiences are distinctive learning opportunities and should be used fully.

Several strategies can support IPE faculty development. For example, we offer an annual in-person IPE training program that brings faculty together across professions, with experienced IPE faculty providing guidance to new faculty who are beginning their IPE work. This training focuses on general interprofessional team facilitation skills, best practices, and strategies for avoiding common pitfalls. One pitfall, for example, is that interprofessional facilitators may talk too much about their own experiences and expertise, preventing students from learning from one another [26].

In addition to annual training, we offer just-in-time training for individual IPE events. For each class in our introductory FICP course, faculty facilitators receive an online facilitator guide that includes recommended preparation. Faculty arrives 30 minutes before the course begins to review the facilitator guide, which describes the activities, expectations and tips for facilitators, key points for discussion and debriefing, and timelines for each learning segment, including theory bursts or short lectures, team discussion questions, simulations or role-plays, and case studies. Just-in-time training provides an efficient way to prepare busy faculty to deliver high-quality team facilitation for IPE activities.

## Faculty recruitment

Faculty representing the student professions participating in each IPE activity should be engaged in that activity. Faculty facilitators are important role models of interprofessional collaboration for students. Faculty from at least two professions deliver theory bursts and instructions for each IPE activity. To reduce hierarchical relationships among professions that are often inherent in health care, all faculty, regardless of academic degree or credentials, are called “Professor” during IPE class time; this practice helps level the playing field.

How to incentivize faculty participation in IPE is another important consideration. Formal recognition at the institutional level, including recognition in job expectations or promotion criteria, is useful [17]. Incentives may differ across health professions programs. For the FICP course, serving as a faculty facilitator is part of the faculty assignment for nursing, pharmacy, and social work. Medicine relies primarily on clinical faculty, many of whom actively practice in the community and count participation as academic service. The FICP course has been offered for 10 years, so we also provide opportunities for third-year pharmacy students and second- or third-year medical students who participated in the course as entry-level students to substitute when faculty facilitators are absent. These students receive credit toward leadership hours for this effort.

## Assessment and evaluation

When individual IPE activities or broader IPE programs are developed, it is important to establish both (1) a plan for assessing learning outcomes and (2) a plan for evaluating program quality for improvement purposes [17]. The IOM proposed a conceptual framework for measuring the impact of IPE that remains relevant today [16]. The Interprofessional Learning Continuum model outlines key considerations for measuring IPE impact, beginning with the context of the learning experience. This model emphasizes IPE as a continuum from education to practice and highlights the need to consider the level and stage of learners participating in the activity.

A second consideration is the selection of appropriate learning outcomes, which may include topic-specific outcomes in addition to relevant IPEC competencies. Kirkpatrick typology is widely used in IPE to categorize outcomes [29]. This framework includes learners’ reactions to the experience (Level 1), changes in attitudes or perceptions (Level 2a), acquisition of knowledge and skills (Level 2b), behavioral change (Level 3), broader changes in organizational practice (Level 4a), and patient or client outcomes (Level 4b). Selected outcomes should align with both the learner’s stage of development and the context of the educational activity.

When possible, educators should use a published or validated IPE instrument that aligns with the intended outcomes. If an existing tool does not fully meet the needs of the activity, adapting a validated instrument may be preferable to developing a new instrument. The National Center for Interprofessional Practice and Education maintains a repository of assessment tools with descriptions of their intended uses and targeted outcomes (Table 1).

In addition to interprofessional competencies, educators may assess the acquisition of knowledge and skills specific to the topic of the learning activity, such as emergency preparedness. These items are typically developed for the activity and should align with the stated learning outcomes. An evaluation process is also helpful for supporting continuous quality improvement. This process involves collecting feedback from students and facilitators to inform revisions to the activity or program.

In the FICP course, we use several approaches to assessment and evaluation. At the end of each module, students complete a brief self-assessment designed to support reflection on the experience. The self-assessment includes items related to topic-specific knowledge and skill development, questions about the quality of the IPE experience, and open-ended prompts. Two open-ended items are evaluative and ask students what supported their learning and what changes they would recommend. This process allows students to reflect on their learning while also providing information to guide course improvement.

To assess changes in interprofessional competencies over time, we use the Interprofessional Collaborative Competency Attainment Survey (ICCAS), a validated instrument focused on collaborative behaviors [30]. The ICCAS is administered once at the end of the course rather than after each module. This approach helps reduce survey fatigue and reflects our view that competency development is better assessed across a series of learning experiences than through a single activity.

## Resources

Interprofessional organizations, such as the National Academies of Practice, have emerged to support the development of interprofessional competencies, education, and collaborative practice. Table 1 lists IPE-focused organizations and selected website resources that can facilitate the design and implementation of required IPE programs, as well as interprofessional faculty and health care provider development. Regional or state educational networks may also provide IPE online resources and opportunities, such as the Texas IPE Consortium’s virtual synchronous course for a Rapid Teaming in Experiential Learning Environments Certificate.

**Figure 1.**
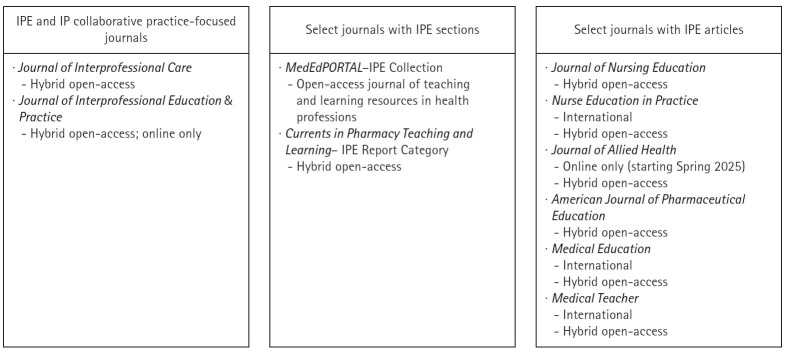
Resources for IPE and IP collaborative practice scholarship. Hybrid open-access means that access to the journal is primarily by paid subscription except when authors pay for immediate open-access to their own articles. IP: Interprofessional; IPE: interprofessional education.

For more than 15 years, IPE has developed as an area of scholarship with journals dedicated to IPE and interprofessional collaborative practice. Education journals from different professions have also published IPE articles (Figure 1). Publications on IPE and interprofessional collaborative practice are rich sources of evidence that can support requirements for IPE and provide data for evaluating specific IPE activities and programs.

In addition, several IPE toolkits are available as resources, including those by Noureddine et al. [6] and the National League for Nursing [10]. TeamSTEPPS 3.0, which includes online curriculum materials, videos, training tools, and checklists, is widely available [21]. Examples of TeamSTEPPS 3.0 content include SBAR, or situation, background, assessment, recommendation/request; closed-loop communication; handoffs; situation monitoring; mutual support; and advocacy and assertion.

## Conclusion

The future of health care depends on well-trained, integrated, highly functioning interprofessional teams dedicated to person-centered care and population health. The IPE community includes many committed educators and health care providers from a broad range of professions who believe that high-quality IPE is needed to improve health care systems, deliver safe and high-quality person-centered care, and improve health care providers’ work-life balance [17]. Preparing future health care providers for effective interprofessional collaborative practice requires sustained, longitudinal IPE experiences that are integrated across curricula and delivered to students in all health professions [17].

Nursing remains central to interprofessional collaboration because nurses are frontline health care professionals who provide most of the direct patient care and improve care quality through care coordination and teamwork [6,10]. Nurses are expected to be full partners on interprofessional teams and to lead changes needed to improve the quality and safety of health care [6,9]. Mandates for IPE within accreditation requirements provide the impetus for nursing and other health care programs to design IPE that equips learners with the competencies required for the teamwork needed to improve care quality.

Many programs encounter challenges in developing required, high-quality IPE that produces collaboration-ready graduates. In this article, we have examined several issues that should be considered when launching IPE programs. We have explored possible solutions, based on evidence and our experience, and highlighted readily available resources that can support the planning and delivery of IPE programs and activities. Although translating IPE into effective interprofessional collaborative practice across all health care settings remains an ongoing endeavor, integrating required IPE into health professions curricula should help develop a cadre of interprofessional clinical leaders who, over time, can shift health care culture further toward interprofessional team-based care.

## Figures and Tables

**Table 1.** IPE organizations and resources

IPE-focused organization	Types of resources
Interprofessional Education Collaborative (IPEC) (https://www.ipecollaborative.org/)	· IPEC Core Competencies documents [7,13]
	· IPEC Institutional Assessment Instrument
	· Faculty Development Institutes for individual or team participants; synchronous and asynchronous learning experiences
	· Poster collection
National Center for Interprofessional Practice and Education (https://nexusipe.org/informing/about-national-center)	· Annual virtual Nexus Summit conference, including posters, lightning talks, seminars, and plenaries
	· NexusIPE Leadership Academy
	· Office of Interprofessional Continuing Professional Development, which offers joint accreditation for activities and programs
	· Guidance on Developing Quality Interprofessional Education for the Health Professions document [17]
	· T3 Train-the-Trainer: Interprofessional Team Development Program, a 3-day synchronous online and project-based training program for teams
	· Assessment & Evaluation: Measurement Instrument Collection
	· Professional Directory
National Academies of Practice (NAP): 17 different academies, including nursing, physician, pharmacy, psychology, and physical therapy (https://www.napractice.org/)	· NAP Telepractice Toolkit: A Resource for Interprofessional Collaborative Practice (2023)
	· Educational Resources on the Social Determinants of Mental Health
	· Annual meeting and forum
	· Cross-academies webinars
Interprofessional Global (https://interprofessional.global/)	· Regional and emerging networks
	· Publications and reports
	· Global Cafes: live webinars for exchanging interprofessional experiences
Agency for Healthcare Research and Quality’s TeamSTEPPS 3.0 [21] (https://www.ahrq.gov/teamstepps-program/index.html)	· Curriculum materials, including modules on communication, team leadership, situational monitoring, and mutual support
	· Diagnosis Improvement Course
	· Training simulation videos and patient videos
	· Measurement tools
	· Research and evidence base
	· Pocket guide

HPAC: Health Professions Accreditors’ Collaborative; IPE: interprofessional education; TeamSTEPPS: Team Strategies & Tools to Enhance Performance and Patient Safety.
